# The Effects of a Curcumin Derivative and Osimertinib on Fatty Acyl Metabolism and Mitochondrial Functions in HCC827 Cells and Tumors

**DOI:** 10.3390/ijms241512190

**Published:** 2023-07-29

**Authors:** Min-Tsang Hsieh, Pei-Chih Lee, Yi-Ting Chiang, Hui-Yi Lin, Der-Yen Lee

**Affiliations:** 1 Drug Development Center, China Medical University, Taichung 406040, Taiwan; t21917@mail.cmu.edu.tw (M.-T.H.); ytchiang@mail.cmu.edu.tw (Y.-T.C.); pingababy@yahoo.com.tw (H.-Y.L.); 2 School of Pharmacy, China Medical University, Taichung 406040, Taiwan; 3 Chinese Medicinal Research and Development Center, China Medical University Hospital, Taichung 40447, Taiwan; 4 Graduate Institute of Biomedical Sciences, China Medical University, Taichung 406040, Taiwan; pclee@mail.cmu.edu.tw; 5 Research Center for Cancer Biology, China Medical University, Taichung 406040, Taiwan; 6 Cancer Biology and Precision Therapeutics Center, China Medical University, Taichung 406040, Taiwan; 7 Pharmacy Department, China Medical University Hsinchu Hospital, Hsinchu Country 302, Taiwan; 8 Graduate Institute of Integrated Medicine, China Medical University, No. 91, Hsueh-Shih Road, Taichung 40402, Taiwan

**Keywords:** tyrosine kinase inhibitor, metabolomics, fatty acyl metabolism, acylcarnitine, carnitine, mitochondrial stress

## Abstract

Drug combination therapy is a key approach in cancer treatments, aiming to improve therapeutic efficacy and overcome drug resistance. Evaluation of intracellular response in cancer cells to drug treatment may disclose the underlying mechanism of drug resistance. In this study, we aimed to investigate the effect of osimertinib, a tyrosine kinase inhibitor (TKI), and a curcumin derivative, 35d, on HCC827 cells and tumors by analyzing alterations in metabolome and related regulations. HCC827 tumor-bearing SCID mice and cultured HCC827 cells were separately examined. The treatment comprised four conditions: vehicle-only, 35d-only, osimertinib-only, and a combination of 35d and osimertinib. The treated tumors/cells were subsequently subjected to metabolomics profiling, fatty acyl analysis, mitochondrial potential measurement, and cell viability assay. Osimertinib induced changes in the ratio of short-chain (SC) to long-chain (LC) fatty acyls, particularly acylcarnitines (ACs), in both tumors and cells. Furthermore, 35d enhanced this effect by further lowering the SC/LC ratio of most ACs. Osimertinib and 35d also exerted detrimental effects on mitochondria through distinct mechanisms. Osimertinib upregulated the expression of carnitine palmitoyltransferase I (CPTI), while 35d induced the expression of heat shock protein 60 (HSP60). The alterations in ACs and CPTI were correlated with mitochondrial dysfunction and inhibited cell growth. Our results suggest that osimertinib and 35d disrupted the fatty acyl metabolism and induced mitochondrial stress in cancer cells. This study provides insights into the potential application of fatty acyl metabolism inhibitors, such as osimertinib or other TKIs, and mitochondrial stress inducers, such as curcumin derivatives, as combination therapy for cancer.

## 1. Introduction

Epidermal growth factor receptor (EGFR)-activating mutations have been targeted for the treatment of non-small cell lung cancer (NSCLC). A prospective study has revealed a significant frequency of EGFR mutations in tumors from Asian patients with adenocarcinoma (746 (51.4%) positive; 704 (48.6%) negative out of 1450 patients) [1,2]. In the case of advanced or metastatic EGFR-mutant NSCLC, EGFR tyrosine kinase inhibitors (TKIs) are the established first-line treatment [3]. Lung cancers harboring EGFR mutations typically exhibit a positive response to EGFR TKIs. However, the development of drug resistance invariably emerges over time. In a cohort of 37 patients with NSCLCs carrying EGFR mutations, all drug-resistant tumors retained their original EGFR-activating mutations. Additionally, some of these tumors acquired well-known mechanisms of resistance, such as the EGFR T790M mutation or MET gene amplification [4]. Osimertinib (also known as AZD9291, tagrisso) is a third-generation EGFR TKI that targets the cysteine 797 residue to form a covalent bond in the ATP-binding site of EGFR kinase. Osimertinib demonstrates high selectivity for EGFR-activating mutations and the EGFR T790M mutation and has been approved as the first-line treatment for patients with the metastatic EGFR T790M mutation-positive NSCLCs [5,6].

Drug resistance is one of the challenges in cancer therapy, and the underlying mechanisms still remains largely unknown. Mitochondria play a crucial role in cancer cell survival in stressful conditions by coordinating multiple characteristics of cells, such as cellular respiration, fatty acid oxidation, the tricarboxylic acid cycle, electron transport, Ca^2+^ signaling, and redox homeostasis [7]. Emerging evidence strongly suggests that the development of drug resistance in cancer cells is certainly associated with mitochondrial-related pathways [8,9,10]. Furthermore, high mitochondrial respiration and oxidative phosphorylation status were observed in resistant tumor cells [11,12]. Consequently, targeting mitochondria for cancer therapy and overcoming drug resistance has garnered increasing attention for various types of cancer [7]. For instance, metformin was observed to inhibit mitochondria complex I and has been repurposed as an anticancer drug. Preclinical and clinical studies have demonstrated its antitumor efficacy in combating resistance caused by chemotherapeutics, including cisplatin, doxorubicin, and 5-fluorouracil (ClinicalTrials.gov Identifier: NCT00897884 and NCT02437656) [13,14,15,16]. In a phase II clinical trial, the combination of metformin with standard EGFR-TKI therapy significantly improved both progression-free survival and overall survival for patients with advanced lung adenocarcinoma (NCT03071705) [17]. Additionally, CPI-613, a lipoate analog that targets two enzyme complexes (α-ketoglutarate dehydrogenase and pyruvate dehydrogenase) in the TCA cycle [18], demonstrated anticancer activity in pancreatic cancer and acute myeloid leukemia (AML) [19]. Notably, CPI-613 has the ability to sensitize AML cells to cytarabine and mitoxantrone, representing a promising approach for relapsed or refractory AML [20]. Two representative compounds, metformin (ClinicalTrials.gov Identifier: NCT04559308) and CPI-613 (ClinicalTrials.gov Identifier: NCT04203160), have advanced to phase III clinical trials. Therefore, the development of treatment targeting mitochondria holds the potential to enhance the precision of cancer treatment at lower drug doses. Additionally, exploring more potent molecules that target mitochondrial functions can be an alternative for achieving successful clinical application in cancer treatment.

Given that curcumin, an active compound from the rhizomes of *Curcuma longa* L. (turmeric), has been recognized for its antioxidant, anti-inflammatory, and anticancer activities, curcumin and its various derivatives have been developed as anticancer agents [21,22,23,24]. However, the unfavorable pharmacokinetics and limited specificity of these compounds restricted the application in cancer therapies. In order to address these challenges, research groups have made efforts to develop curcumin-loaded nanoparticles, which have demonstrated promising results in terms of efficient drug loading and selective targeting of tumor cells [25]. Previously, we developed a series of curcumin derivatives and evaluated their anticancer potential. Among these derivatives, bis(hydroxymethyl) alkanoate curcuminoid derivatives exhibited in vitro antiproliferative and in vivo antitumor activities [26,27,28,29]. In our investigations, we identified 35d, a propargyl modified bis(hydroxymethyl) alkanoate curcuminoid, as particularly effective in inhibiting the proliferation of HCC827 cells. Furthermore, we discovered that combining 35d with osimertinib resulted in significant enhancement of the anticancer activity of both compounds when used in combination.

In order to further investigate the effect of 35d with osimertinib, we conducted metabolomics profiling to elucidate the changes provoked by these compounds. The results revealed that fatty acyls, especially acylcarnitines (ACs), were the predominant class of metabolites altered in tumor tissues from 35d- and osimertinib-treated mice. Specifically, we observed that osimertinib induces an imbalance in the ratio of short-chain to long-chain fatty acyls, with a notable effect on ACs. The carnitine pool, consisting of L-carnitine and its acylated derivatives, i.e., ACs, is recognized for facilitating fatty acid oxidation (FAO) in mitochondria and peroxisomes [30,31]. L-carnitine and the ACs are closely tied to components of mitochondrial function and have the potential to serve as biomarkers for illness and drug response, including adverse drug reactions (ADRs) [32]. Many ADRs are caused by the toxicity of off-target drug interactions with mitochondria, and impairment of mitochondrial FAO is associated with an accumulation of fatty acid derivatives such as ACs in plasma and urine [33,34,35]. Thus, the effects of osimertinib and 35d on cancer cells may also involve their impact on mitochondrial function. To further explore the effects of osimertinib and 35d on mitochondria, we investigated the influence on mitochondrial function and structure. Both compounds interfere with the mitochondrial function and morphology through distinct mechanisms. The administration of osimertinib and 35d, both in vivo and in vitro, resulted in similar changes in the levels of L-carnitine and ACs in tumor tissues and cultured HCC827 cells. Specifically, osimertinib led to the accumulation of long-chain ACs and depletion of short-chain ACs. On the other hand, 35d specifically induced the expression of heat shock protein 60 (HSP60), which is a mitochondrial stress protein [36,37]. Additionally, 35d enhanced the osimertinib-induced accumulation of long-chain ACs. These findings shed light on the effect of osimertinib on mitochondrial metabolism and highlighted the potential anticancer applications of 35d.

## 2. Results

### 2.1. Alteration of Fatty Acyl Metabolism in the Tumors from Osimertinib- and 35d-Treated Mice

**Figure 1:**
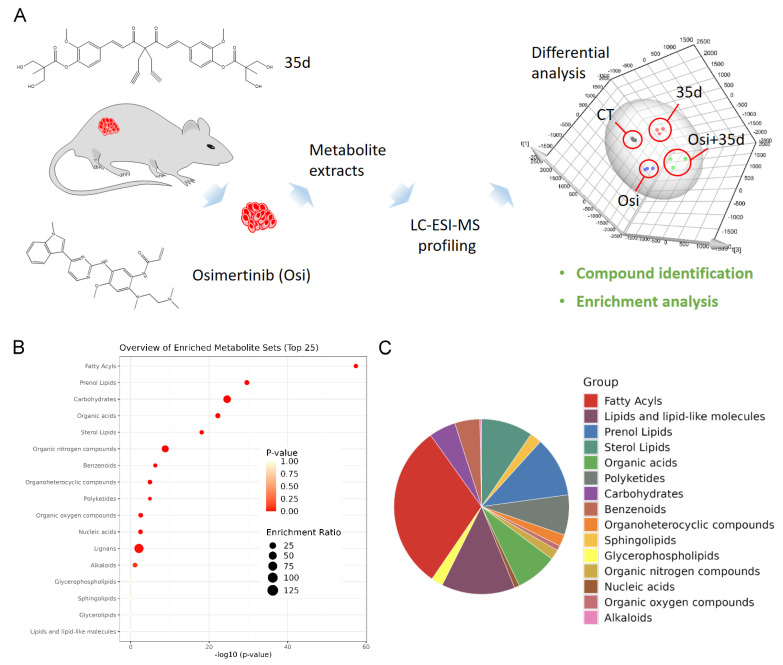
Non-targeting metabolomics profiling to reveal the metabolites affected by osimertinib and 35d. (**A**) Overview of the procedure used to identify differentially expressed metabolites in tumors from osimertinib- and 35d-treated mice. (**B**) Enrichment analysis to classify the differentially expressed metabolite targets in the tumors from osimertinib- and 35d-treated mice. (**C**) The distribution of differentially expressed metabolite targets across chemical compound superclasses. Osi: Osimertinib; Osi + 35d: Osimertinib + 35d.

Tumors from control mice, mice treated with 35d alone, osimertinib alone, and a combination of osimertinib and 35d were collected for metabolite extraction, followed by LC-ESI-MS analysis. The LC-MS data were analyzed to identify differentially expressed metabolites, which were further classified using enrichment analysis (Figure 1A). The LC-MS signals that exhibited differential grouping in the principal component analysis (PCA) were selected for compound identification, resulting in a dataset of differentially expressed metabolites among the treatment groups. The dataset was further subjected to enrichment analysis for classification. As a result, fatty acyl, prenol lipids, carbohydrates, organic acids, and sterol lipid were categorized as the top five sets of enriched metabolites (Figure 1B). Among the 434 targeted metabolites, 99 were classified as fatty acyls, 44 as lipids and lipid-like molecules, and 36 metabolites as prenol lipids. Based on these findings, we hypothesized that one of the potent effects of osimertinib and 35d could be the modulation of tumor proliferation through alterations in fatty acyl metabolism.

### 2.2. Accumulation of Long-Chain Free Fatty Acids in the Tumors with Osimertinib Treatment

**Figure 2:**
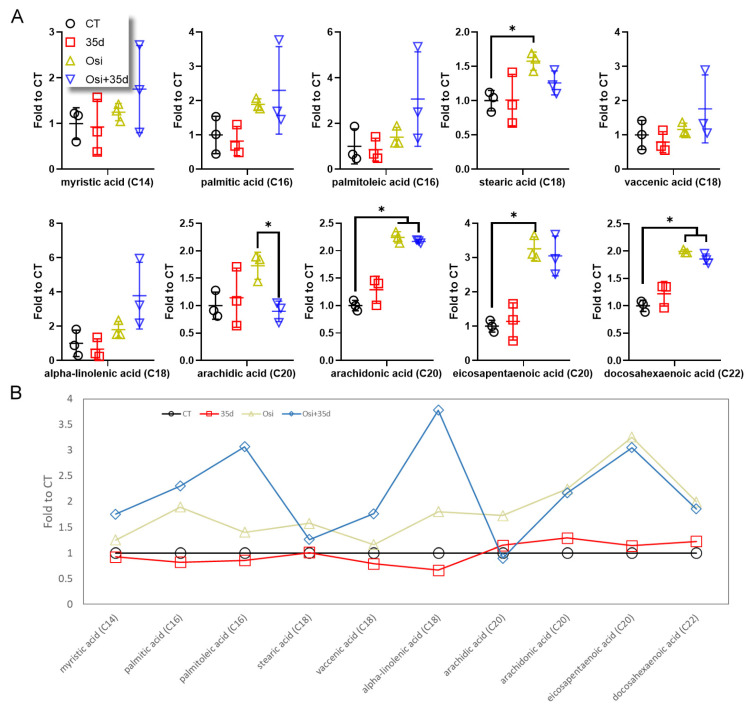
The level of common free fatty acids in tumors from 35d- and osimertinib-treated mice. (**A**) The level of detected common fatty acids (C14 to C22) in the tumor tissues. (**B**) The mean levels of the fatty acids in the four groups of tumors (*n* = 3, * indicates *p* < 0.05). Osi: Osimertinib; Osi + 35d: Osimertinib + 35d.

To investigate the fatty acyl metabolism, we initially targeted common free fatty acids and analyzed 10 targets with saturated and unsaturated hydrocarbon chains ranging from 14 to 22 carbons (Figure 2 and Figure S1). Among these targets, stearic acid, arachidonic acid, eicosapetanenoic acid, and docodahexaenoic acid exhibited a significant increase in the tumors from osimertinib-treated mice (Figure 2A). While the treatment with osimertinib showed a tendency to cause accumulation of other detected fatty acids, statistical significance was not achieved (Figure 2A,B). Notably, treatment with 35d alone did not significantly affect the levels of these fatty acids (Figure 2A,B). However, when osimertinib and 35d were combined, myristic acid, palmitic acid, palmitoleic acid, vaccenic acid, and alpha-linolenic acid displayed higher mean values in the tumors (Figure 2B). These findings indicate that osimertinib treatment can impact fatty acid metabolism, and the presence of 35d can further enhance these changes, although 35d alone does not independently cause these alterations.

### 2.3. The Osimertinib-Induced Accumulation of Long-Chain Acylcarnitines and Depletion of Short-Chain Acylcarnitines Were Enhanced by 35d in Tumors

**Figure 3:**
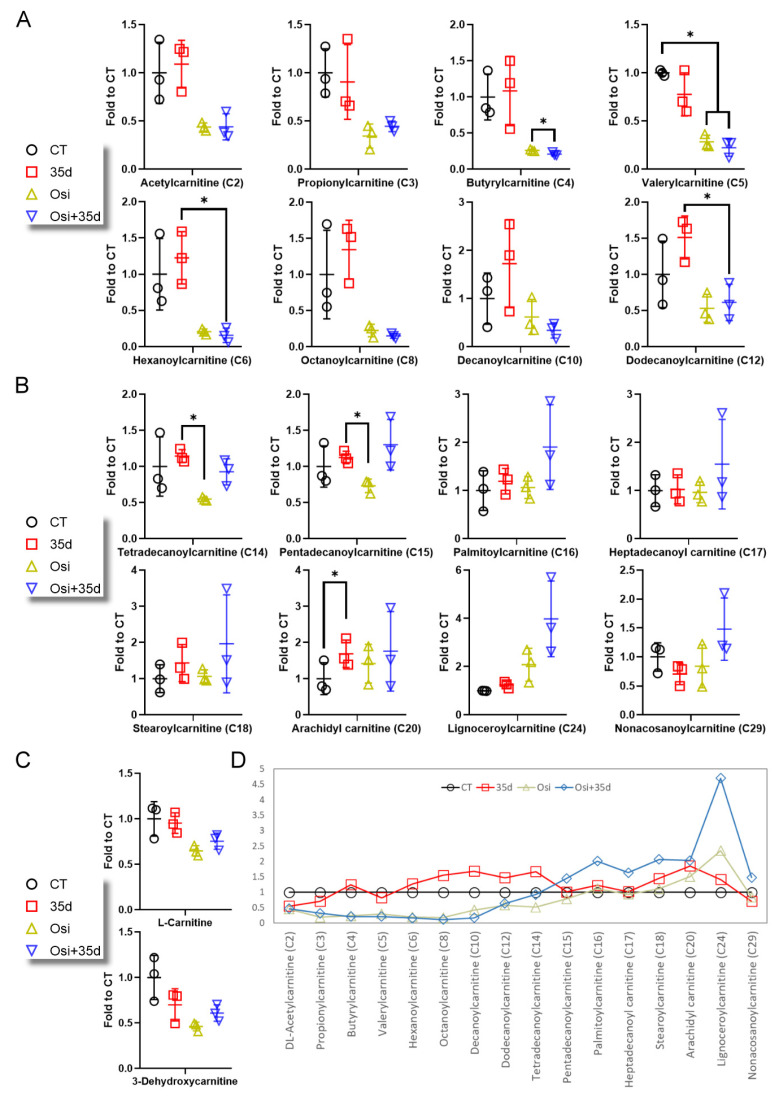
The level of carnitines and acylcarnitines (ACs) in tumors from 35d- and osimertinib-treated mice. (**A**) The level of detected ACs of C2 to C12 in the tumors. (**B**) The level of detected ACs of C14 to C29 in the tumors. (**C**) The level of L-carnitine and 3-dehydroxycarnitine in the tumors. (**D**) The mean levels of the ACs in the four groups of tumors (*n* = 3, * indicates *p* < 0.05). Osi: Osimertinib; Osi + 35d: Osimertinib + 35d.

To further investigate fatty acyl metabolism, we examined the levels of a series of acylcarnitines (ACs) and L-carnitine (Figure 3 and Figure S2). ACs ranging from C2 to C12 were measured, and in general, the levels of these ACs were lower in tumors from osimertinib- and Osi + 35d-treated mice. However, treatment with 35d alone did not significantly affect the levels of these ACs compared to the control group (Figure 3A). Among the ACs with chain length greater than C14, osimertinib did not significantly induce the accumulation of long-chain ACs. Conversely, the treatment with 35d resulted in an increase in certain long-chain ACs, such as arachidyl carnitine, compared to the control group (Figure 3B). Notably, the Osi + 35d treatment led to the highest mean levels of most long-chain ACs, except for tetradecanoylcarnitine (C14) and arachidyl carnitine (C20) (Figure 3B). Overall, the levels of free carnitines (L-carnitine and 3-dehydroxycarnitine) in tumors were lower in mice treated with 35d alone, osimertinib alone, and the Osi + 35d combination (Figure 3C). When considering the collective data, we observed that the levels of ACs resulting from 35d alone were similar to those of the control group (Figure 3D). However, treatment with osimertinib led to a decrease in the mean levels of short-chain ACs and an increase in the mean levels of long-chain ACs. Importantly, the presence of 35d further enhanced the accumulation of long-chain ACs induced by osimertinib, resulting in the highest mean levels in tumors (Figure 3D). These results demonstrate that the metabolism of free fatty acid and ACs in tumors can be influenced by osimertinib, and the effect of osimertinib can be augmented by 35d.

### 2.4. The Effect of Osimertinib and 35d on Acylcarnitine Metabolism in HCC827 Cells 

**Figure 4:**
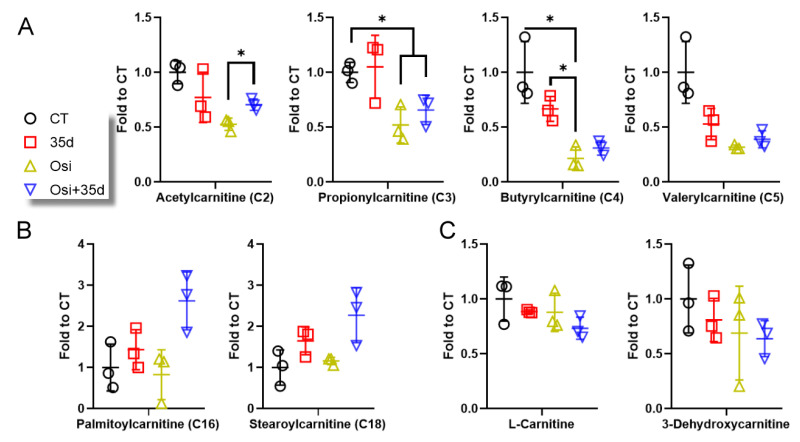
The level of carnitines and ACs in 35d- and osimertinib-treated HCC827 cells. (**A**) The level of detected ACs of C2 to C5 in the cells (*n* = 3). (**B**) The level of detected ACs of C16 and C18 in the cells. (**C**) The level of L-carnitine and 3-dehydroxycarnitine in the cells (*n* = 3, * indicates *p* < 0.05). Osi: Osimertinib; Osi + 35d: Osimertinib + 35d.

To further investigate the impact of osimertinib and 35d, HCC827 cells were treated by 35d, osimertinib, and the Osi + 35d combination, and the cellular carnitines and ACs were monitored by LC-ESI-MS analysis (Figure 4 and Figure S3). The treatment with osimertinib resulted in a decrease in short-chain ACs, regardless of the presence of 35d (Figure 4A). In terms of long-chain ACs, treatment with osimertinib alone did not show any significant difference in the level of palmitoylcarnitine (C16) and stearoylcarnitine (C18) compared to the control group (Figure 4B). However, the treatment with the Osi + 35d combination led to an increase in the levels of palmitoylcarnitine (C16) and stearoylcarnitine (C18) (Figure 4B). L-carnitine and 3-dehydroxycarnitine levels slightly decreased in the osimertinib- and 35d-treated HCC827 cells (Figure 4C). These results align with the findings observed in tumors and cultured cells, indicating a similar change in the homeostasis of ACs. Specifically, osimertinib treatment depletes short-chain ACs, and the Osi + 35d combination further enhances the accumulation of long-chain ACs (Figure 3 and Figure 4). Thus, the depletion of short-chain ACs and accumulation of long-chain ACs in tumors and cells with osimertinib and 35d treatment may be associated with mitochondrial stress or damage and impaired fatty acyl metabolism.

### 2.5. The Effect of Osimertinib and 35d on Mitochondria in HCC827 Cells

**Figure 5:**
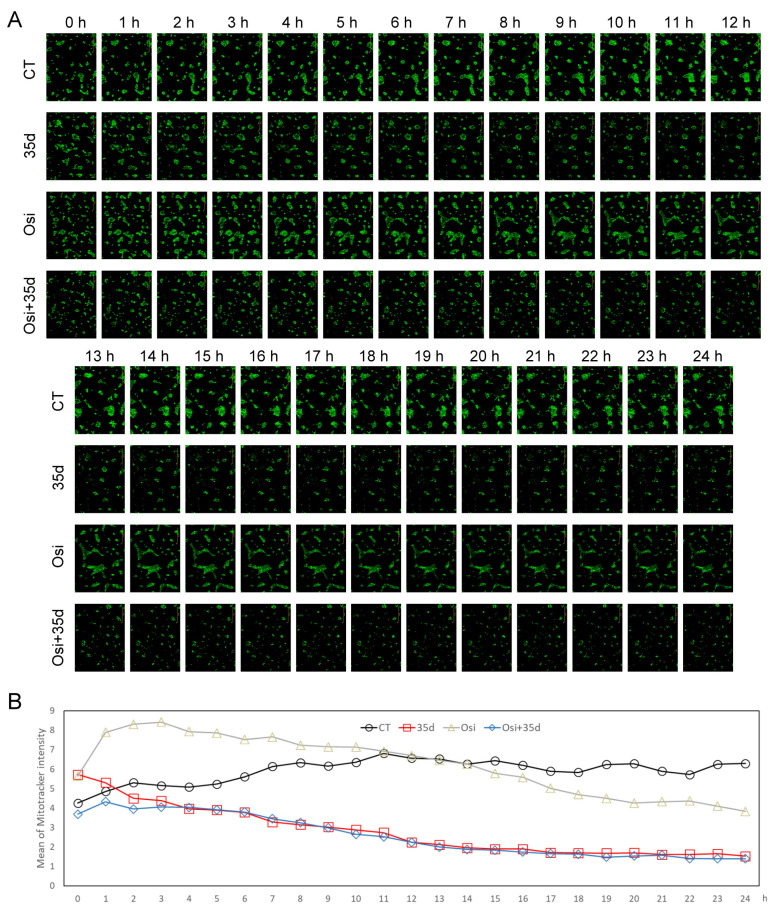
Real-time monitoring of mitochondria in osimertinib- and 35d-treated cells. (**A**) The acquired images of MitoTracker fluorescence in osimertinib- and 35d-treated cells with a timeline spanning 24 h. (**B**) Quantification of MitoTracker fluorescence intensity from the images. Osi: Osimertinib; Osi + 35d: Osimertinib + 35d.

To further investigate the changes in mitochondria, HCC827 cells were administered with MitoTracker and then exposed to 35d, osimertinib, and the Osi + 35d combination, and then the cells were cultured and monitored by real-time fluorescence microscopy. The fluorescence intensity of MitoTracker was monitored over time in the control cells and cells treated with 35d, osimertinib, and the Osi + 35d combination (Figure 5A). The mean fluorescence intensity of MitoTracker was quantified using ImageJ software 1.52a and analyzed. As a result, the control cells showed a 1.5-fold increase in MitoTracker fluorescence intensity after 7 h of incubation compared to the initial measurement. In the 35d-treated cells, the MitoTracker fluorescence intensity decreased to 50% after 10 h of incubation and remained at around 30% after 15 h of incubation. In the osimertinib-treated cells, the MitoTracker fluorescence intensity increased to 1.5-fold after 3 h of incubation but gradually decreased to 70% after 24 h of incubation. The cells treated with the Osi + 35d combination exhibited a similar trend to 35d-treated cells but with a weaker MitoTracker intensity during the initial 0–3 h interval (Figure 5B). These results indicate that both osimertinib and 35d can induce changes in mitochondrial distribution and reduce the mitochondrial mass in the treated HCC827 cells. 

### 2.6. The Effect of Osimertinib and 35d on the Expression of Mitochondrial Proteins in HCC827 Cells

**Figure 6:**
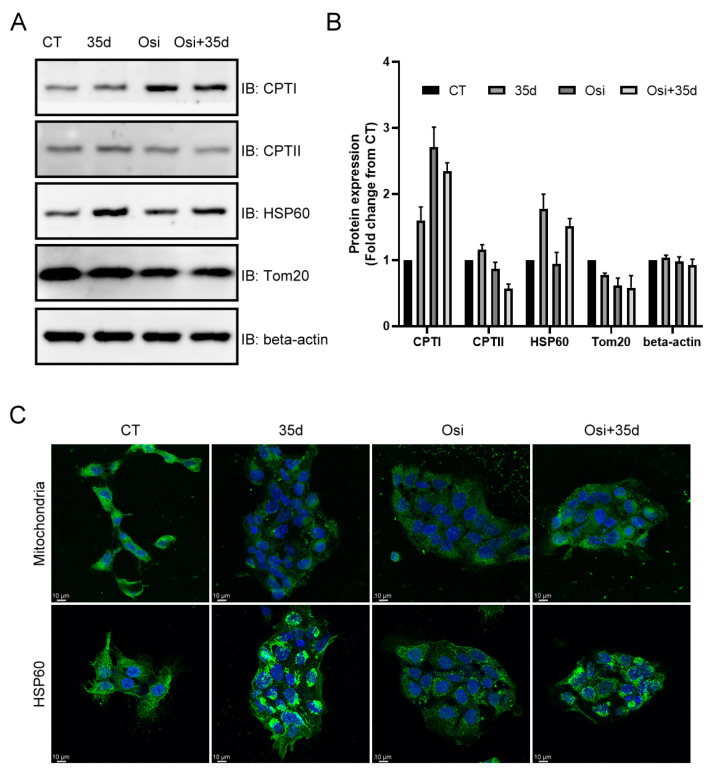
The expression and distribution of mitochondrial proteins in 35d- and osimertinib-treated HCC827 cells. (**A**) The immunoblots of mitochondrial proteins, (**B**) the quantitative data for immunoblots (*n* = 3), and (**C**) the confocal images of MitoTracker staining and immunofluorescence for HSP60 were acquired from the HCC827 cells with indicated treatment. Osi: 0.25 μM osimertinib; 35d: 2.5 μM 35d; Osi + 35d: 0.25 μM osimertinib + 2.5 μM 35d.

To further investigate the effects of osimertinib and 35d on mitochondria, we examined the expression of mitochondrial proteins in the HCC827 cells treated with 35d, osimertinib, and the Osi + 35d combination. Immunoblots were performed, and the results showed that the expression of Tom20, a mitochondrial protein, was decreased in cells treated with 35d, osimertinib, and the Osi + 35d combination (Figure 6A,B). Treatment with osimertinib, both with and without the presence of 35d, resulted in the upregulation of CPTI, a mitochondrial protein involved in fatty acid metabolism (Figure 6A,B). The treatment with 35d induced the increased expression of CPTI, CPTII, and HSP60, while the expression of CPTII was reduced by the presence of osimertinib (Figure 6A,B). Notably, the treatment of 35d led to the increased expression of HSP60, even in the presence of osimertinib (Figure 6A,B). Confocal images of MitoTracker fluorescence revealed a decrease in intensity and a diffused distribution of mitochondria in cells treated with osimertinib, 35d, and the Osi + 35d combination. Notably, the treatment with osimertinib altered the distribution of HSP60 without affecting its expression (Figure 6). In contrast, the treatment with 35d decreased the intensity of MitoTracker fluorescence and resulted in a condensed distribution of HSP60 around the peripheral area of nuclei, regardless of the presence of osimertinib (Figure 6C). Overall, these findings indicate that both 35d and osimertinib can modify the composition and structure of mitochondria to different states. 

### 2.7. The Curcumin-Derived Compound 35d as a Mitochondrial Stress Inducer to Enhance Osimertinib’s Anticancer Properties

**Figure 7:**
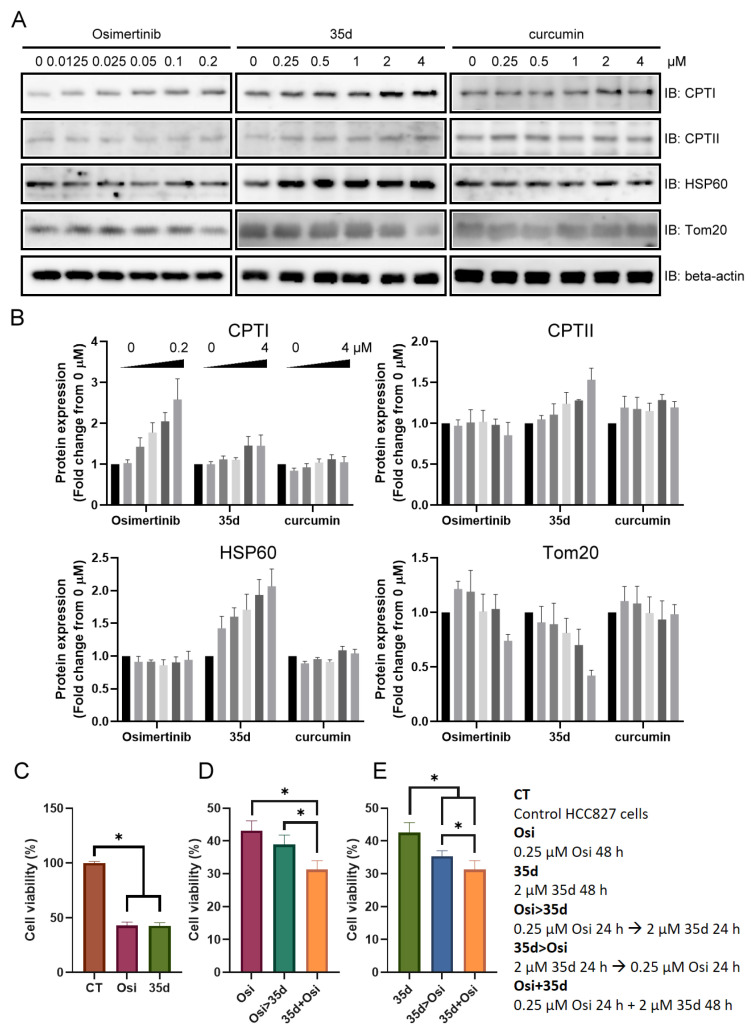
The effect of osimertinib and 35d on HCC827 cells. (**A**) The immunoblots of mitochondrial proteins and (**B**) the quantitative data for immunoblots (*n* = 3) were acquired from the HCC827 cells treated with the indicated concentrations of osimertinib, 35d, and curcumin. The cell viability of osimertinib- and 35d-treated HCC827 cells with different combinations compared with (**C**) the control cells and (**D**) osimertinib-treated cells and (**E**) the 35d-treated cells (*n* = 6, * indicates *p* < 0.05).

Next, we compared the effect of osimertinib, 35d, and curcumin on mitochondria and investigated the potential of 35d in combination with osimertinib for cancer treatment. We observed that the expression of CPTI increased in response to the increasing osimertinib concentrations, while the expression of Tom20 decreased at higher osimertinib concentrations. Conversely, increasing 35d concentrations led to increased expression of CPTI, CPTII, and HSP60 and decreased expression of Tom20. Curcumin, on the other hand, did not significantly affect the expression of these mitochondrial proteins (Figure 7A,B). These results suggest that osimertinib affects mitochondria by interfering with the CPTI- and CPTII-related metabolism, while 35d induces mitochondrial stress. 

Based on these differential effects on mitochondria, we further examined the combination of 35d and osimertinib in cancer treatment. HCC827 cells treated with 0.25 μM osimertinib or 2 μM 35d showed similar viability, approximately 43% (Figure 7C). When HCC827 cells were treated with osimertinib for 24 h, followed by 35d for another 24 h, or vice versa, the viability decreased to 39% and 35%, respectively (Figure 7D,E). Finally, the combined treatment with 0.25 μM osimertinib and 2 μM 35d resulted in a viability of 31% (Figure 7E). These results suggest that 35d can impair the mitochondrial function by inducing mitochondrial stress, and in combination with osimertinib, it can enhance osimertinib-induced dysregulation of fatty acyl metabolism. Therefore, 35d has potential as an anticancer agent by targeting mitochondria and potentiating the effects of osimertinib. 

### 2.8. Lowering the Short-Chain/Long-Chain Acylcarnitine Ratio as a Feature of Mitochondrial Metabolic Dysfunction

**Figure 8:**
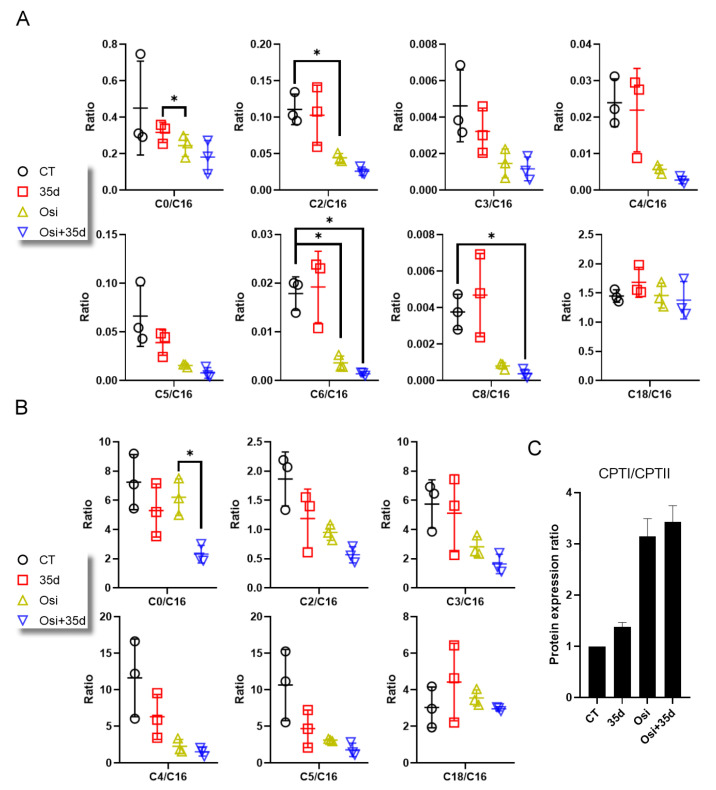
The SC/LC ratio of ACs and carnitine palmitoyltransferases (CPTs). (**A**) The ratio of L-carnitine (C0) or ACs (C2–C6, C8, and C18) to palmitoylcarnitine (C16) in the tumors. (**B**) The ratio of L-carnitine (C0) or ACs (C2–C5 and C18) to palmitoylcarnitine (C16) in the HCC827 cells. (**C**) The expression ratio of CPTI to CPTII in the HCC827 cells (*n* = 3, * indicates *p* < 0.05). Osi: Osimertinib; Osi + 35d: Osimertinib + 35d.

The upregulation of CPTI could facilitate the accumulation of long-chain ACs (LCACs) and affect the acylcarnitine transfer and catabolism of fatty acyls in mitochondria. Our findings indicate that the treatment of 35d induced the increased expression of CPTI and CPTII. Furthermore, the upregulation of HSP60 induced by 35d suggests its ability to induce mitochondrial stress. For evaluating the homeostasis of ACs in tumors from osimertinib- and 35d-administered mice, we assessed the ratio of short-chain ACs (SCACs) to an LCAC (palmitoylcarnitine; C16). According to the results, the SC/LC ratios of most ACs but not the LC/LC ratio, C18/C16, were generally lower in tumors from osimertinib-administered mice, while the presence of 35d had no significant effect on these ratios. However, a substantially lower SC/LC ratio of most ACs was also determined in tumors from the Osi + 35d-administered mice (Figure 8A). Similar trends were observed in HCC827 cells, where osimertinib led to a general reduction in the SC/LC ratio of most ACs, while 35d exhibited a moderate effect on the ratios of C4/C16 and C5/C16. Notably, a substantially lower SC/LC ratio of ACs was also determined in the Osi + 35d-treated cells (Figure 8B). The decreased SC/LC ratio of ACs suggests impaired acyl transfer mediated by CPTI and CPTII. Subsequent analysis of the CPTI/CPTII ratio confirmed their elevation in response to 35d and osimertinib treatment (Figure 8C). Thus, the decreased SC/LC ratio of ACs is associated with altered CPTI and CPTII expression, highlighting mitochondrial dysfunction in the context of mitochondrial metabolism.

## 3. Discussion

In this study, we initiated a metabolomics study to assess the effect of a TKI, osimertinib, and a curcuminoid derivative, 35d, on transplanted tumors. Through LC-ESI-MS metabolomics profiling, we discovered that osimertinib induces an imbalance in the ratio of short-chain to long-chain fatty acyls, particularly ACs. Furthermore, the effect of osimertinib on ACs is further amplified by the addition of 35d in both tumors and cultured cells. Subsequently, we examined the change in mitochondria and identified that both osimertinib and 35d inflict damage on mitochondria through distinct mechanisms. Our study reveals, for the first time, that osimertinib exerts an influence on metabolism of ACs by inducing the upregulation of CPTI. Notably, 35d induces the upregulation of HSP60 and enhances the osimertinib activity. Moreover, the reduction in SC/LC ratios of most ACs following osimertinib treatment may be associated with CPTI and CPTII expression, indicating mitochondrial metabolism dysfunction. Therefore, our findings present an alternative perspective in TKI cancer therapy by considering different aspects, such as mitochondrial dysfunction in metabolism and mitochondrial stress response.

The primary anticancer activity of osimertinib is to disrupt tyrosine kinase signaling. However, our findings suggest that osimertinib also affects the mitochondrial functions. Metabolomics profiling revealed that fatty acyl metabolites in tumors appeared to be the dominant affected species upon administering osimertinib (Figure 1). Treatment of mice with osimertinib plus 35d resulted in notable changes in the compositions of free fatty acids and ACs in tumors, specifically leading to the accumulation of long-chain fatty acids and long-chain ACs (Figure 2 and Figure 3). While the administration of 35d alone in mice did not exert a significant effect on fatty acyls, it noticeably enhanced the metabolic changes induced by osimertinib, particularly augmenting the accumulation of certain long-chain fatty acids and most long-chain ACs (Figure 2 and Figure 3). These results indicate that the effect of 35d on mitochondria differs from that of osimertinib, yet 35d is potent enough to enhance the action of osimertinib. Although there is no evidence supporting that drug resistance can develop by reversing the osimertinib-induced mitochondrial impairment, we hypothesize that cancer cells may have capability to recover from or adapt to the change in mitochondrial metabolism. From this perspective, combining 35d with osimertinib or other TKIs may have the potential to reinforce their effect on mitochondria and may prove beneficial in treatment strategies. 

Although the ACs detected in cultured HCC827 cells were fewer than those in transplanted tumors, the change in L-carnitine and ACs levels in the osimertinib- and 35d-treated cells resembled that in tumors from osimertinib- and 35d-administered mice, specifically a decrease in short-chain ACs and an increase in long-chain ACs (Figure 4). Since L-carnitine and the ACs can reflect a certain mitochondrial condition, such as an accumulation of fatty acid derivatives in ADRs [32,33,34,35], the activity of osimertinib and 35d in cancer cells may also stem from their impact on mitochondria. Indeed, real-time monitoring of mitochondria in HCC827 cells revealed the deterioration of mitochondria by the assaults of osimertinib and 35d (Figure 5). Moreover, the suppression of Tom20 indicated the action of osimertinib and 35d on mitochondria (Figure 6A,B), and the confocal image illustrated the change in mitochondria provoked by the treatment (Figure 6C). Interestingly, certain mitochondrial proteins increased in response to the treatment. Osimertinib alone or combined with 35d notably upregulated CPTI, while 35d specifically induced the upregulation of HSP60 (Figure 6A,B). The increase in CPTI levels could be a direct consequence of osimertinib, as a compensatory mechanism to remove the accumulation of long-chain ACs. In contrast, the induction of HSP60, a mitochondrial stress marker, by 35d suggests its potential to activate the mitochondrial unfolded protein response (UPR^mt^) [36]. Although only 35d caused the upregulation and condensed distribution of HSP60 around the peripheral area of nuclei, osimertinib also altered the distribution of HSP60 without affecting the expression (Figure 6C). Thus, we propose that 35d and osimertinib can modify mitochondrial structure and function by different mechanisms. 

Based on the changes observed in mitochondrial proteins induced by osimertinib, 35d, and curcumin, osimertinib and 35d have distinct effects on the expression of mitochondrial proteins, while curcumin does not exhibit significant activity (Figure 7A,B). Evaluation of 35d- and osimertinib-induced cell death revealed an IC_50_ of approximately 0.25 μM and 2 μM, respectively, with the conditions of 35d > Osi and 35d + Osi treatments yielding a more pronounced effect (Figure 7C–E). These results demonstrate that cancer cells primed with 35d become susceptible to osimertinib, indicating that mitochondrial stress may potentiate osimertinib-induced dysregulation of fatty acyl metabolism. Resistance to cell death is a hallmark of cancer, and a number of lipid metabolism pathways have been revealed to induce cancer cell death [38]. The disturbance of carnitine and ACs in the treatment with TKIs has been noticed and considered as having a possible connection to metabolism of and toxicity to organs [39]. From an LC-MC/MS profiling to examine differences between subpopulations of osimertinib persister cells, principal component analysis (PCA) showed that over 229 quantified metabolites can be used to distinguish the cycling persisters, non-cycling persisters, and untreated populations. The results indicated that higher abundance of acylcarnitine species in cycling persister cells and the increase in FAO over time with osimertinib treatment both suggest that mitochondrial FAO may contribute to the cycling persister phenotype [40]. Consequently, the lower SC/LC ratio of ACs in treated cells or tumors likely originates from the primary effect of osimertinib on wild type HCC827 cells (Figure 8A,B). Since CPTI and CPTII are essential for fatty acyl transfer and FAO in mitochondria [32], the increase in CPTI/CPTII ratio in response to 35d and osimertinib treatment likely contributed to the lower SC/LC ratio of ACs (Figure 8C). As cells acquire drug resistance, persister cells may recover from the loss of FAO function and maintain a higher abundance of AC species.

Mitochondrial activity is crucial to meet the high energy demands of cancer cells, correspondingly resulting in an accumulation of mitochondrial reactive oxygen species (mtROS) and mutations in mitochondrial DNA in cancer cells. Therefore, the UPR^mt^ becomes essential for tumor growth and progression, with HSP60 playing an essential role in the regulation of these stresses. While several reports suggest a positive function of HSP60 in tumor growth [36,37], its role in the regulation of apoptosis appears to be more complex. The role of cytosolic HSP60 in the regulation of apoptosis is contradictory, as it has both a prosurvival function and participates in the apoptosis process triggered by cell death stimuli [41,42,43,44]. Additionally, the reduction of HSP60 in mice affects adipose tissue homeostasis, leading to alterations in body weight, body composition, and adipocyte morphology, albeit exhibiting local insulin resistance [45]. Hence, the role of HSP60 extends beyond the regulation of UPR^mt^ and cell survival functions. Our results showed that osimertinib and 35d could induce the redistribution of HSP60 in the treated HCC827 cells, with 35d even upregulating HSP60. However, the specific interaction between mitochondrial stress response and apoptosis during the action of osimertinib remained unclear. Notably, 35d can be classified as a UPR^mt^ inducer, and importantly, it induced mitochondrial stress that leads to cell death and potentiated the anticancer properties of osimertinib. Therefore, compounds with the ability to induce UPR^mt^ may exhibit similar activity, and developing or screening compounds targeting UPR^mt^ induction could be a feasible strategy to discover a new therapeutic approach for treating cancers. 

## 4. Materials and Methods

### 4.1. Tumor Samples from Drug-Treated Mice

SCID mice purchased from Lasco Co., Ltd. (Taiwan) were used in the study. All experiments were supervised under the Institutional Animal Care and Use Committee, China Medical University, Taichung, Taiwan with a protocol number CMUIACUC-2020-060. HCC827 cells were inoculated subcutaneously into the hind limbs of SCID mice. The synthesis of 35d was initiated from curcumin via esterification with 2,2,5-trimethyl-1,3-dioxane-5-carboxylic acid to afford an ester intermediate, which was in turn di-alkylated with alkyl bromide and hydrolyzed with HCl (aq) [26]. When the tumor volume reached 200–250 mm^3^, mice were randomized into four groups (*n* = 3, per group) and treated with vehicle (5% ethanol and 10% Tween 80 in 85% saline, 0.1 mL), 35d (100 mg/kg), osimertinib (1 mg/kg), or the combination of 35d (100 mg/kg) and osimertinib (1 mg/kg), respectively. The compounds were administered orally on a daily basis for eight consecutive days. At the end of experiments, mice were sacrificed and tumor nodules were dissected for the subsequent study. 

### 4.2. Metabolite Sample Preparation

The tumor samples were homogenized as 1 mg of tissue in 2 μL of ultrapure water using 1 mm zirconium oxide grinding beads and then centrifuged at 14,000× *g* for 10 min. Each supernatant was mixed with 4 volumes of 100% methanol and kept at −80 °C for 2 h. Then, the mixture was centrifuged at 14,000× *g* for 10 min, and 45 μL of supernatant from methanol extraction was mixed with 45 μL of ultrapure water. After centrifugation at 14,000× *g* for 10 min, the supernatant was transferred into an insert vial pending LC-ESI-MS analysis. 

For HCC827 cells, 1 × 10^6^ cells were lysed in 100 μL of ultrapure water by sonication for 5 min, twice. The lysate was centrifuged at 14,000× *g* for 10 min, and 100 μL of cell lysate was mixed with 400 μL of 100% methanol and kept at −80 °C for 2 h. After centrifugation at 14,000 rpm for 10 min, 500 μL of supernatant from methanol extraction was transferred to a new microtube for vacuum drying. Each dry metabolite sample was dissolved in 50 μL of 50% methanol and subjected to centrifugation at 14,000× *g* for 10 min. The supernatant from each dissolved sample was then transferred to an insert vial pending LC-ESI-MS analysis.

### 4.3. Liquid Chromatography–Mass Spectrometry

The LC-MS system consisted of an ultraperformance LC system (ACQUITY UPLC I-Class, Waters, Milford, MA, USA) and an electrospray ionization/atmospheric pressure chemical ionization (ESI/APCI) source of 4 kDa quadrupole time-of-flight mass spectrometer (Waters VION, Waters). A BEH C18 column (2.1 × 100 mm, Walters, Milford, MA, USA) was used for chromatographic separation. The flow rate was set to 0.2 mL/min, with a column temperature of 35 °C, and each sample was subjected to LC-MS with a 7.5 μL sample injection. The sample contents were eluted using 60% mobile phase A (ultrapure water + 0.1% formic acid) and 40% mobile phase B (100% methanol + 0.1% formic acid). Subsequently, the contents were held at 40% B for 0.5 min, raised to 95% B for 5.5 min, held at 95% B for 1 min, and then lowered to 40% B for 1 min. The column was equilibrated by pumping 40% B for 4 min. The chromatogram and spectrum were acquired using ESI+ and ESI- modes under the following conditions: capillary voltage of 2.5 kV, source temperature of 100 °C, desolvation temperature of 250 °C, cone gas maintained at 10 L/h, desolvation gas maintained at 600 L/h, and acquisition in MS^E^ mode with a range of 100–1000 m/z and a 0.5 s scan time.

### 4.4. LC-MS Data Processing

The profiling data were acquired using UNIFI 1.8 software (Waters, Milford, MA, USA) and processed using Progenesis QI 2.1 software with EZinfo (Waters, Milford, MA, USA). The LC-MS signals acquired in ESI+ and ESI- modes were uploaded to Progenesis QI with EZinfo for data normalization, peak picking, compound measurement, and statistical analysis. The LC-MS signals were then converted to datasets by using Progenesis QI. The datasets were subjected to statistical and pathway analysis by applying the MetaboAnalysis 5.0 tool (https://www.metaboanalyst.ca/MetaboAnalyst/ accessed on 14 September 2022). The level of each metabolite was estimated by the peak area of the chromatogram for each identified metabolite using UNIFI 1.8 software (Waters, Milford, MA, USA).

### 4.5. Protein Sample Preparation

First, 1 × 10^6^ cells were added with 100 μL of RIPA buffer (Apolo Biochemical Inc, Hsinchu, Taiwan) and disrupted by sonication for 5 min, twice. The cell lysate was obtained by collecting the supernatant from lysed cells with centrifugation at 14,000× *g* for 10 min. The supernatant was mixed with SDS sample buffer containing 200 mM DTT and then subjected to SDS-PAGE and immunoblotting.

### 4.6. Immunoblotting

Proteins in the resolved SDS-PAGE gel were transferred onto a PVDF membrane by electrophoretic transfer. The PVDF membrane was blocked with 3% skim milk in PBS for 1 h, and then the target proteins were respectively probed by anti-CPTI (E-7), anti-CPTII (G-5), anti-HSP60 (H-1), and anti-Tom20 (29) antibody (Santa Cruz biotechnology, Dallas, TX, USA) in 1% BSA overnight at 4 °C. After washing with PBS thrice, each for 10 min, the membrane was further incubated in horseradish peroxidase-conjugated second antibody (PerkinElmer, Waltham, MA, USA) for 1 h. The membrane was washed with PBS three times, each for 10 min, and the signals were detected with the standard ECL protocol (PerkinElmer, Waltham, MA, USA).

### 4.7. Confocal Laser Scanning Microscopy (CLSM) Observation of the Mitochondria and Heat Shock Protein within Cells

To observe the mitochondria, HCC827 cells (1 × 10^5^ cells) were seeded on coverslips, placed in a 6-well plate, and incubated at 37 °C with 5% CO_2_ supply. Once the cells were attached, the reagents including osimertinib, 35d, and the combination of the two drugs were independently treated. At 24 h post-treatment, the reagents were removed, and the cells were washed with PBS twice. Subsequently, the cells were co-incubated with 200 nM of the fluorescent dye MitoTracker™ Green FM (Invitrogen, Thermo Fisher Scientific Inc., Waltham, MA, USA) at 37 °C for 45 min. After removing the excess fluorescent dye and washing the cells with PBS twice, the cells were fixed with 4% formaldehyde for 20 min. Following two additional washes with PBS, the cells were mounted with DAPI (Abcam PLC, Cambridge, UK)-containing mounting medium to simultaneously stain the nucleus. The fluorescence of the mitochondria and cellular nucleus was observed with confocal laser scanning microscopy (CLSM) (Leica TCS SP8, Leica Inc., Wetzlar, Germany) with an excitation wavelength of 488 and 405 nm, respectively, and an emission wavelength of 520 nm, using appropriate filters.

The distribution of HSP60 in HCC827 cells was also observed using CLSM. Prior to observation, the cells were incubated on coverslips placed in a 6-well plate at 37 °C with 5% CO_2_ supply until they attached. The cells were independently treated with osimertinib, 35d, and the combination of the two drugs for 24 h. Afterwards, the cells were fixed with 4% formaldehyde for 20 min. After washing with PBS three times, the cells were permeabilized with 0.5% Triton X-100 (Sigma-Aldrich, subsidiary of Merck KGaA, Burlington, MA, USA) for 30 min at 25 °C. Following another three washes with PBS, the cells were blocked with 3% bovine serum albumin (BSA; Sigma-Aldrich, subsidiary of Merck KGaA, Burlington, MA, USA))/PBS at 25 °C. One hour later, the blocking solution was removed, and the cells were washed with PBS and then incubated with anti-HSP60 antibody solution (diluted 100-fold with 3% BSA/PBS solution) at 4 °C overnight. Following three washes with PBS, the cells were stained with 10 μg/mL antimouse secondary antibody conjugated with Alexa Fluor 488 (Invitrogen, Thermo Fisher Scientific Inc., Waltham, MA, USA) for 1 h at 25 °C. After washing with the PBS, the cells on the coverslips were mounted with the DAPI-containing mounting medium. The fluorescence of the HSP60 and cell nucleus was observed using CLSM with the excitation wavelength of 488 and 405 nm and the emission wavelength of 520 nm, using appropriate filters.

### 4.8. Real-Time Fluorescent Detection of the Mitochondria within Cells

HCC827 cells (1 × 10^3^ cells) were seeded in each well of a 96-well plate. Once the cells were attached, the cells were incubated with 200 nM of the MitoTracker™ Green FM dye for 45 min. Subsequently, the cells were washed with PBS and respectively incubated with osimertinib, 35d, and the combination of the two drugs at 37 °C with 5% CO_2_ supply. The fluorescence of the mitochondria in the cancer cells was detected every hour using the IncuCyte S3 System (Essen BioScienc Inc., Ann Arbor, MI, USA). 

### 4.9. Statistics

All experiments were performed at least three times. The protein levels were determined by quantifying each signal of immunoblots using ImageJ (National Institutes of Health). Statistical comparisons were analyzed using GraphPad Prism software version 8.0.1 (GraphPad Software, San Diego, CA, USA) with one-way ANOVA and multi-comparison tests, as well as unpaired *t*-tests. Significance was considered at a probability error (*p*) < 0.05, and all *p*-values were two-tailed. The plots were created using means with standard deviation for error bars.

## 5. Conclusions

The response of cancer cells to treatment may contain useful information for elucidating the molecular mechanism of drug resistance and evaluating the therapeutic effect. Our findings may provide an alternative perspective in TKI cancer therapy by considering different aspects, such as mitochondrial dysfunction in metabolism and mitochondrial stress response. However, the mechanisms behind the activity of 35d and the effect of osimertinib on fatty acyl metabolism remain unclear. The interacting proteins of 35d are likely key factors in its function, and applying molecular docking or affinity pulldown assays may help identify the potential candidates and further analyze the structure–activity relationship. It is also important to uncover the regulation of fatty acyl metabolism and CPTs in mitochondria affected by osimertinib. Exploring off-target effects and the link to EGFR signaling may provide more insights to address these questions.

## Data Availability

The data presented in this study are available in the article.

## Supplementary Materials

The following supporting information can be downloaded at: https://www.mdpi.com/article/10.3390/ijms241512190/s1.

## Abbreviations

AC: acylcarnitine; ADR: adverse drug reaction; AML: acute myeloid leukemia; CLSM: confocal laser scanning microscopy; CPTI: carnitine palmitoyltransferase I; CPTII: carnitine palmitoyltransferase II; EGFR: epidermal growth factor receptor; FAO: fatty acid oxidation; HSP60: heat shock protein 60; LC: long chain; LCAC: long-chain acylcarnitine; LC-MS: liquid chromatography–mass spectrometry; NSCLC: non-small cell lung cancer; Osi: osimertinib; Osi + 35d: osimertinib + 35d; PCA: principal component analysis; SC: short chain; SCAC: short-chain acylcarnitine; TKI: tyrosine kinase inhibitor; UPR^mt^: mitochondrial unfolded protein response.
