# Supplementary material for: The Effects of a Curcumin Derivative and Osimertinib on Fatty Acyl Metabolism and Mitochondrial Functions in HCC827 Cells and Tumors
Source: Int J Mol Sci. 2023 Jul 29;24(15):12190. doi: 10.3390/ijms241512190 (PMC10418893; doi:10.3390/ijms241512190)
Supplement: Supplementary file 1 [file ijms-24-12190-s001.zip › ijms-2498506-supplementary.pdf]

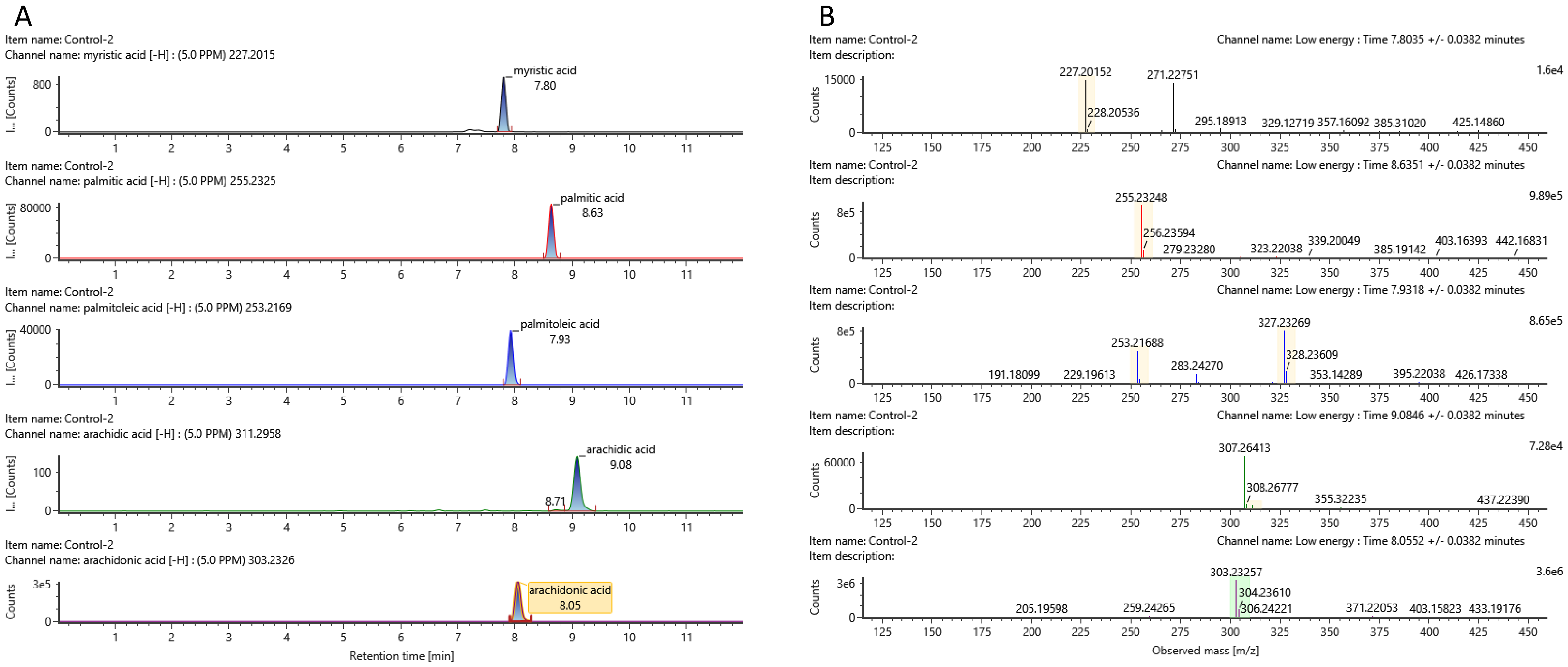

Figure S1. Representative (A) chromatogram and (B) mass spectrum for Figure 2.

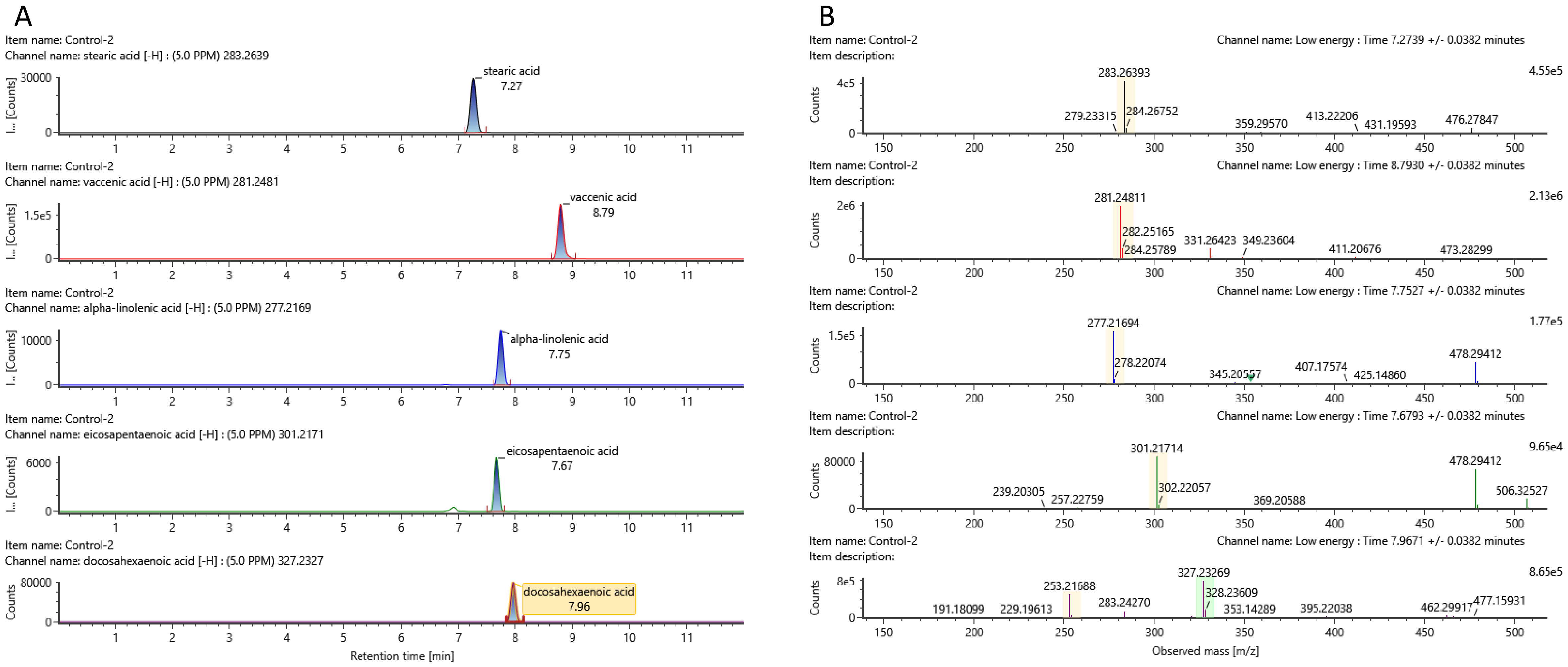

Figure S1. Representative (A) chromatogram and (B) mass spectrum for Figure 2 (continued).

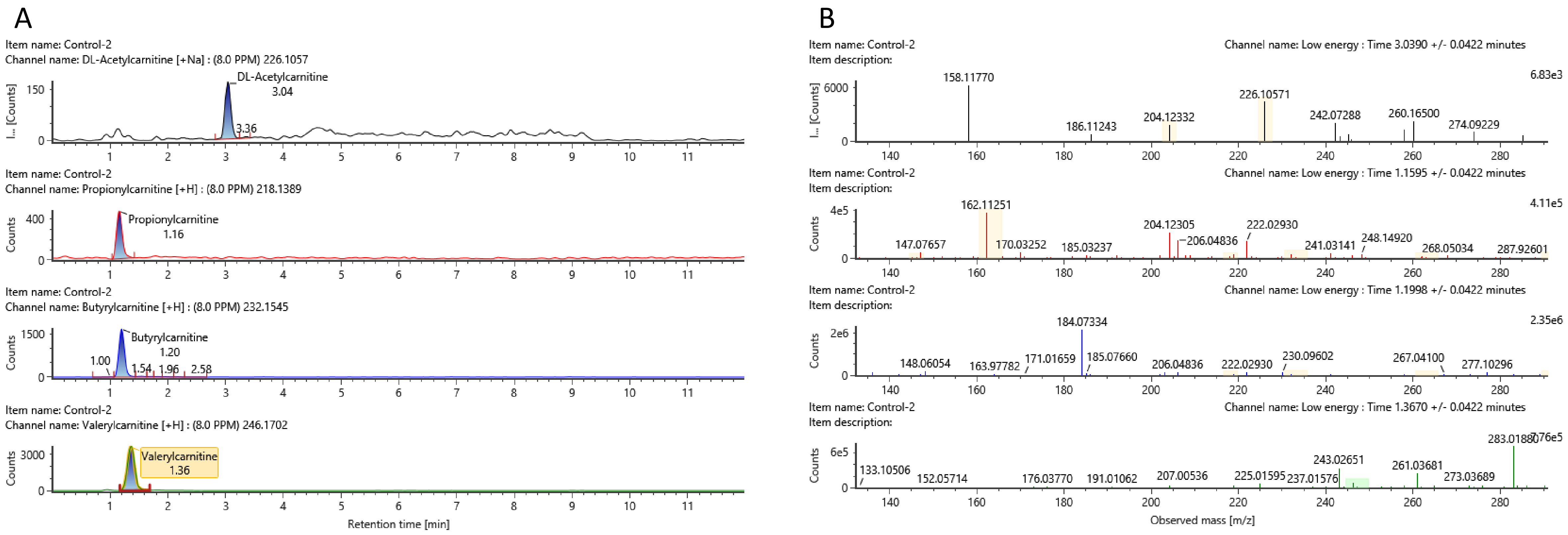

Figure S2. Representative (A) chromatogram and (B) mass spectrum for Figure 3A.

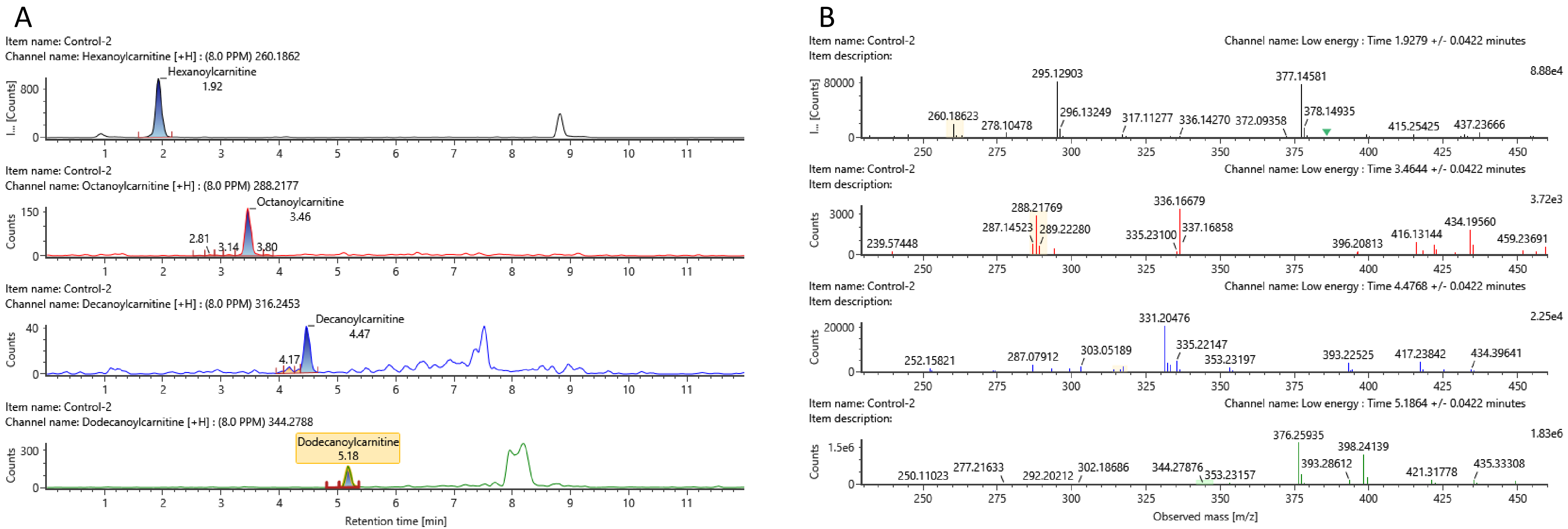

Figure S2. Representative (A) chromatogram and (B) mass spectrum for Figure 3A (continued).

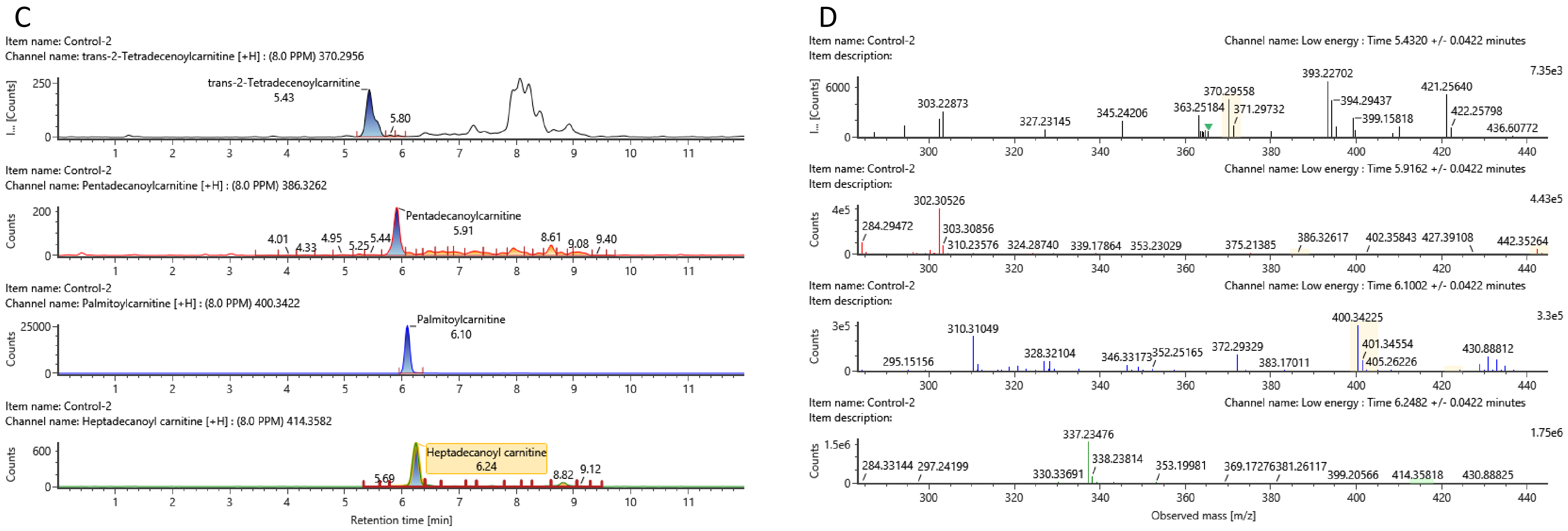

Figure S2. Representative (C) chromatogram and (D) mass spectrum for Figure 3B.

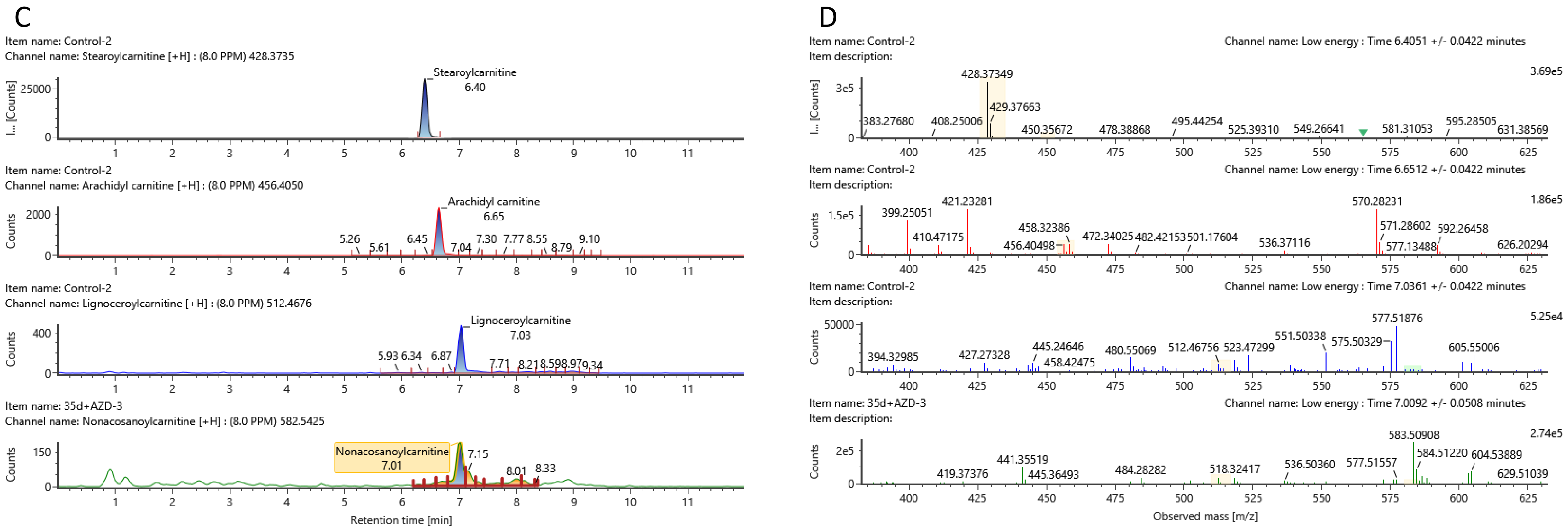

Figure S2. Representative (C) chromatogram and (D) mass spectrum for Figure 3B (continued).

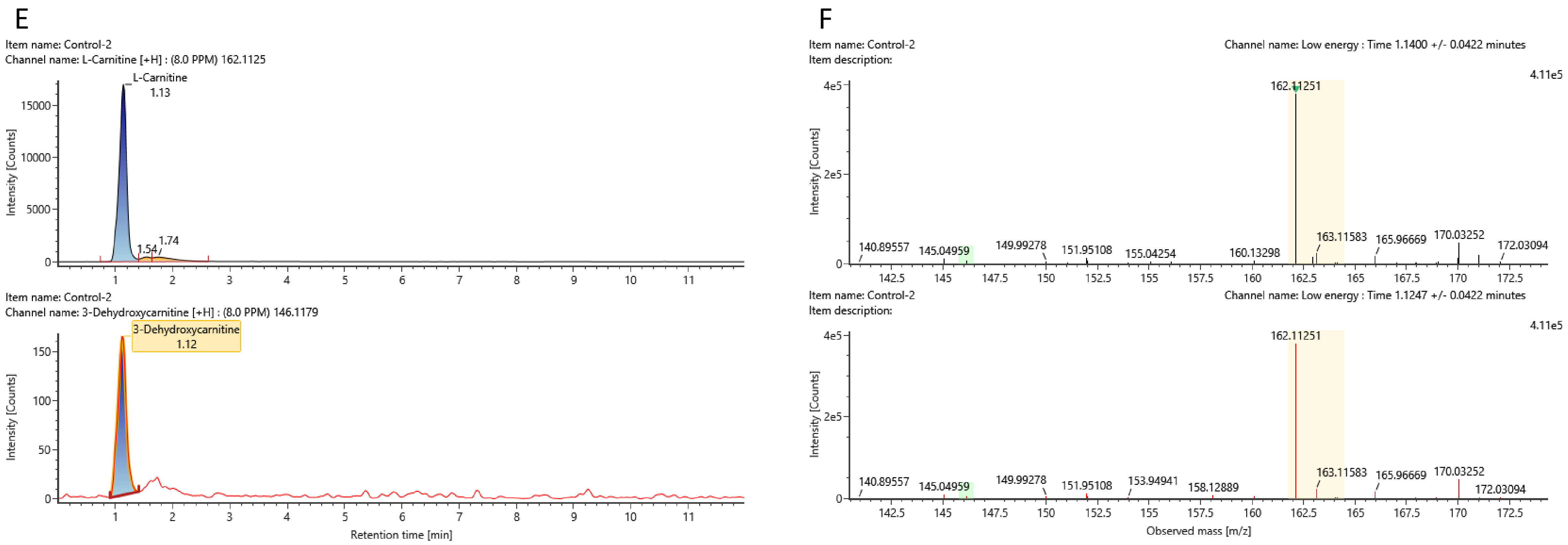

Figure S2. Representative (E) chromatogram and (F) mass spectrum for Figure 3C.

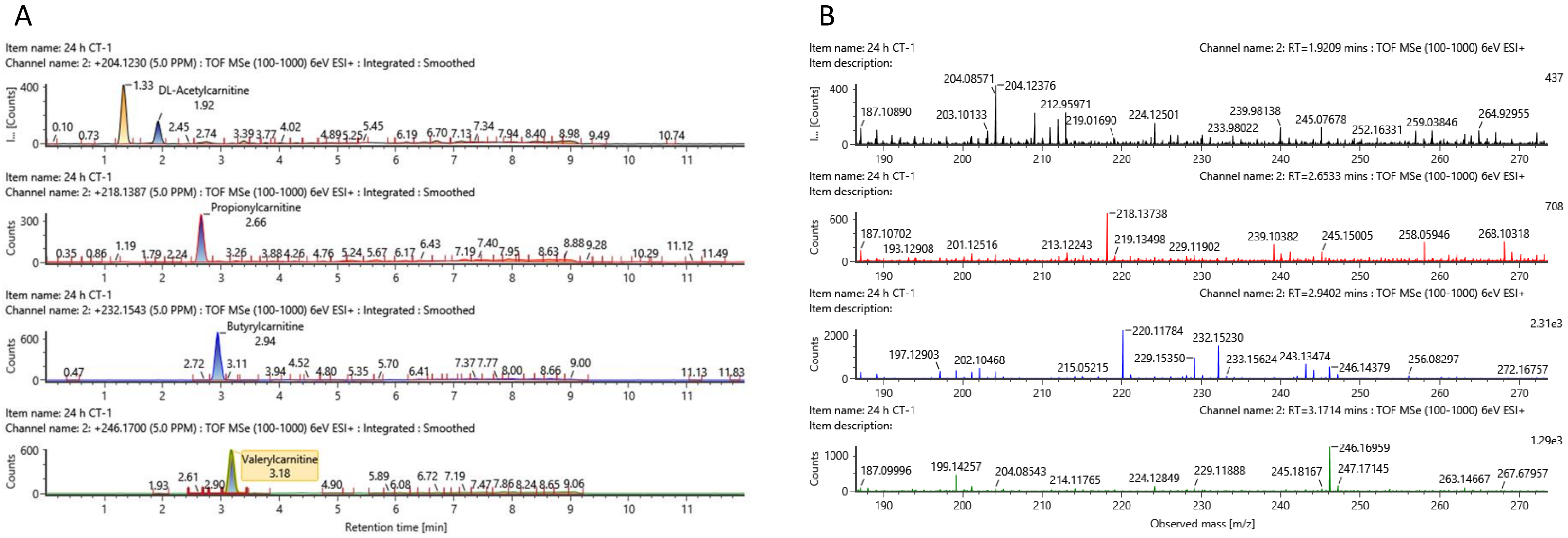

Figure S3. Representative (A) chromatogram and (B) mass spectrum for Figure 4A.

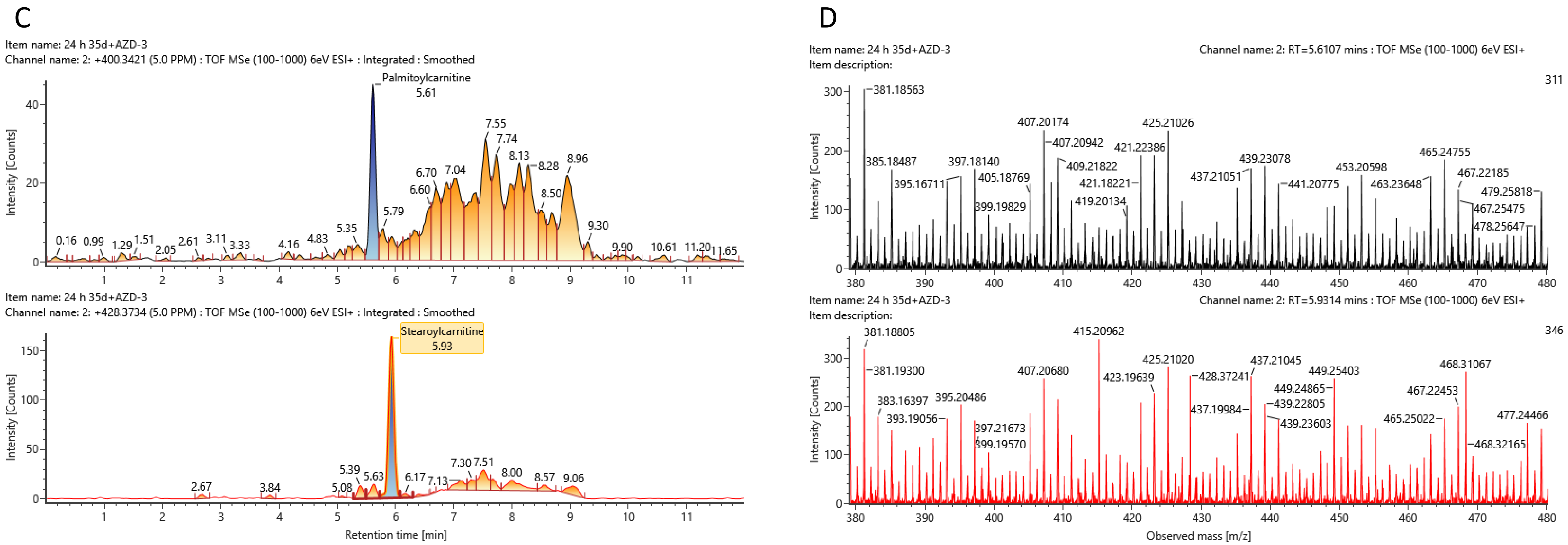

Figure S3. Representative (C) chromatogram and (D) mass spectrum for Figure 4B.

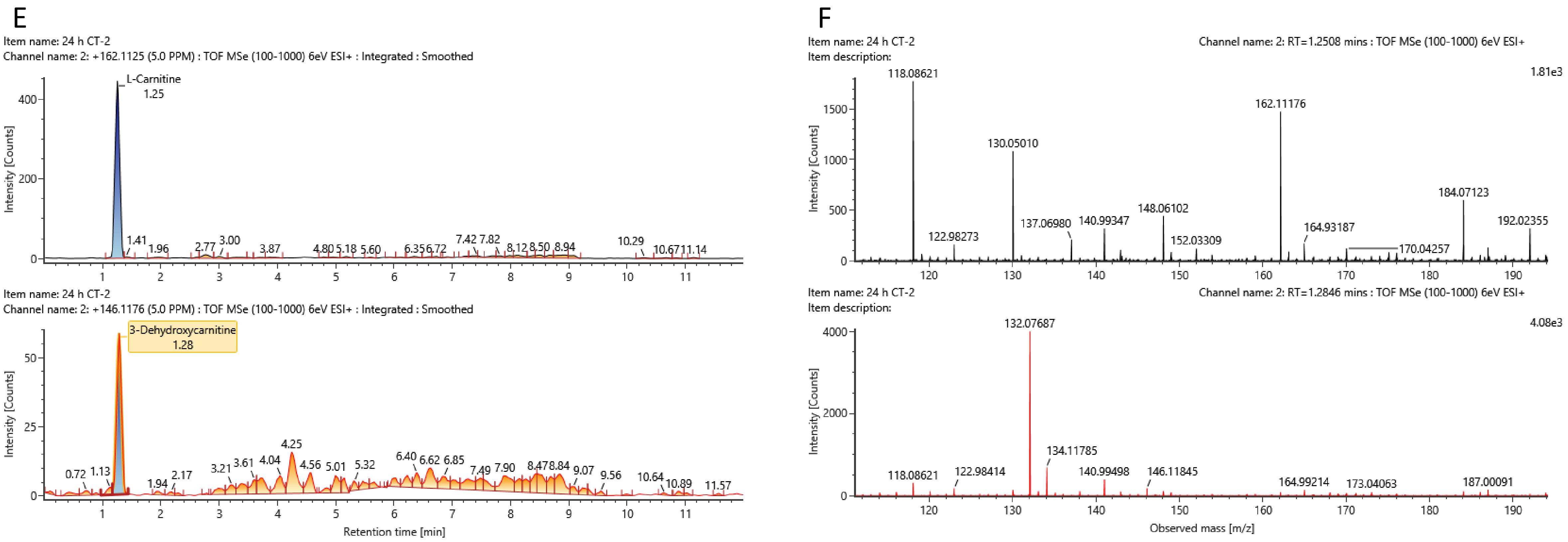

Figure S3. Representative (E) chromatogram and (F) mass spectrum for Figure 4C.
